# Planar or Biaxial Stretching of Poly(ethylene terephthalate) Fiber Webs Prepared by Laser-Electrospinning

**DOI:** 10.3390/ma15062209

**Published:** 2022-03-17

**Authors:** Tomoki Tokuda, Ryo Tsuruda, Takuya Hara, Zongzi Hou, Haruki Kobayashi, Katsufumi Tanaka, Wataru Takarada, Takeshi Kikutani, Juan P. Hinestroza, Joselito M. Razal, Midori Takasaki

**Affiliations:** 1 Faculty of Materials Science and Engineering, Kyoto Institute of Technology, Matsugasaki, Sakyo-ku, Kyoto 606-8585, Japan; tmk.edhf@gmail.com (T.T.); alterguitarist23@gmail.com (R.T.); karuata.bb.0124@gmail.com (T.H.); d9871502@edu.kit.ac.jp (Z.H.); haruki@kit.ac.jp (H.K.); ktanaka@kit.ac.jp (K.T.); 2 Department of Materials Science and Engineering, Tokyo Institute of Technology, 2-12-1 Ookayama, Meguro-ku, Tokyo 152-8550, Japan; takarada.w.aa@m.titech.ac.jp; 3 School of Materials and Chemical Technology, Tokyo Institute of Technology, 4259-J3-142, Nagatsuta-cho, Midori-ku, Yokohama 226-8503, Japan; kikutani@kit.ac.jp; 4 Center for Fiber and Textile Science, Kyoto Institute of Technology, Matsugasaki, Sakyo-ku, Kyoto 606-8585, Japan; 5 Department of Fiber Science and Apparel Design, Cornell University, 135 Human Ecology Building, Ithaca, NY 14850, USA; jh433@cornell.edu; 6 Institute for Frontier Materials, Deakin University, Geelong, VIC 3216, Australia; joselito.razal@deakin.edu.au

**Keywords:** poly(ethylene terephthalate), melt electrospinning, ultrafine fibers, birefringence, crystallinity, planar stretching, biaxial stretching

## Abstract

In this work, laser-heated electrospinning (LES) process using carbon dioxide laser was explored as an eco-friendly method for producing ultrafine fibers. To enhance the thinning of fibers and the formation of fiber structure, planar or equibiaxial stretching and subsequent annealing processes were applied to poly(ethylene terephthalate) (PET) fiber webs prepared by LES. The structure and properties of the obtained webs were investigated. Ultrafine fiber webs with an average diameter of approximately 1 μm and a coefficient of variation of 20–25% were obtained when the stretch ratios in the MD (machine direction) × TD (transverse direction) were 3 × 1 and 3 × 3 for the planar and equibiaxial stretching, respectively. In the wide-angle X-ray diffraction analysis of the web samples, preferential orientation of crystalline c-axis were confirmed along the MD for planar stretching and only along the web plane for equibiaxial stretching, which was in contrast to the stretching of film samples, where additional preferential orientation of benzene ring along the film plane proceeded. The results obtained suggest that PET fiber webs fabricated through LES and subsequent planar or biaxial stretching processes have potential for a wide variety of applications, such as packaging and battery separator materials.

## 1. Introduction

Spun bonding, melt blowing, and electrospinning are widely used to produce nonwoven fabrics. In particular, the electrospinning method has been actively used to form nonwoven materials with submicron- and nanoscale fibers [1,2,3,4,5,6,7,8,9,10,11,12,13,14,15,16,17,18]. Electrospinning can be classified into solution and melt types. Melt electrospinning has the advantages of lower toxicity and cost reduction compared to conventional solution electrospinning [16]. In melt electrospinning, electric heaters and carbon dioxide lasers are mainly used as heat sources [7,8,9,10,11,12,13,14,15,16,17,18].

In previous studies from our group, the melt-electrospinning technique with CO_2_ laser irradiation, i.e., laser-heated electrospinning (LES), was used to investigate the formation of submicron- and nanoscale fibers [10,11,12,13,14,15,17,18]. We applied LES to thermoplastic polymeric materials, such as poly(L-lactide) (PLLA), PLLA copolymers, and PLLA-based composites, to generate webs consisting of relatively uniform fibers with an average diameter and coefficient of variation (CV) of approximately 700–1000 nm and less than 50%, respectively [15,17].

More recently, we prepared the webs of poly(ethylene terephthalate) (PET) fibers through the LES and subsequent annealing at 116 °C for 5 min [18]. The as-spun PET webs exhibited a unique noncrystalline structure with extremely high orientation. After the annealing of the webs, a further increase in the degree of orientation and crystallinity was confirmed through birefringence, wide-angle X-ray diffraction (WAXD) and differential scanning calorimetry (DSC) measurements. It is expected that further thinning of fibers and development of highly ordered fiber structure in the web can be achieved by applying stretching and subsequent annealing to the PET webs obtained by LES.

Many studies are available in the scientific literature on planar and biaxial stretching of PET films for the development of products with highly ordered structure [19,20,21,22,23,24,25,26,27,28], and products of stretched films have been used in many applications, such as packaging sheets [19,22,24,27]. For PET fiber webs, there is a possibility of widening applications, such as packaging and battery separator materials, by applying the stretching process because of their superior mechanical and thermal properties. However, fabrication of ultrafine PET fiber webs through planar or biaxial stretching of the webs from electrospinning has not yet been reported.

In this study, planar or equibiaxial stretching and subsequent annealing processes were applied to poly(ethylene terephthalate) (PET) fiber webs prepared through LES. The structure and properties of the obtained webs were investigated. Similar analyses were performed for planarly or equibiaxially stretched and subsequently annealed PET films for comparison purpose.

## 2. Materials and Methods

### 2.1. Materials

The PET polymer pellets (UNITIKA Ltd., MA-2103) had an intrinsic viscosity of 0.68 dL/g and contained a small amount of TiO_2_.

### 2.2. Laser-Heated Electrospinning (LES) Process

Melt-spun PET fibers (fiber diameter: 151 ± 8 μm (CV: 5%)) were supplied as raw fibers. The melt-spinning conditions of the raw fibers were identical to those reported in the literature [18]. The PET fiber webs were prepared using an LES system (NEU-010, Katotech Co., Ltd., Kyoto, Japan) with a CO_2_ laser light source (PIN-30R, Onizca Glass Co., Ltd., Oume, Japan). Details of the LES apparatus have been reported in a previous publication [18]. The thinning behavior of the fiber in the process was observed using a charge-coupled device (CCD) camera equipped with a telecentric lens with 2× magnification and a personal computer with the software WinROOF 2015 (MITANI Corp., Fukui, Japan). The laser power and applied voltage needed to be varied between 16 and 22 W and 11 and 24 kV, respectively, to stabilize the spin line near the nozzle. The adjustment was performed based on in situ observation of the thinning behavior of the protruded raw fibers.

### 2.3. Planar and Biaxial Stretching Processes

**Figure 1:**
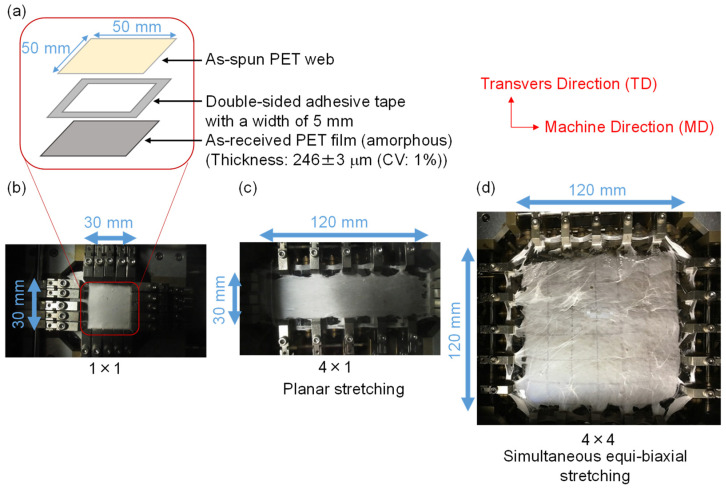
(**a**) Schematic for the preparation of web sample by stretching. Photographs of the samples in the oven (**b**) before stretching, i.e., MD (machine direction) × TD (transverse direction) = 1 × 1, and after planar and simultaneous equibiaxial stretching with the stretch ratios of (**c**) MD × TD = 4 × 1, and (**d**) MD × TD = 4 × 4.

A schematic for sample preparation and photographs of the planar and simultaneous equibiaxial stretching processes are shown in Figure 1. After preparation of PET webs via LES, a web specimen with dimensions of 50 mm × 50 mm and an area density of approximately 5 mg/m^2^ was placed on an amorphous PET film of the same size. The thickness of the film was 246 ± 3 μm (CV: 1%). The web and film were adhered using a double-sided adhesive tape with a width of 5 mm around the outer rim of the film (Figure 1a,b). The prepared sample was set to the biaxial film stretching equipment (18B4, Imoto Machinery, Co., Ltd., Kyoto, Japan). The net sample size, excluding the portion for chucking, was 30 mm × 30 mm in size. Subsequently, the temperature of the oven containing the film stretching system was increased at a rate of approximately 3 °C/min up to a stretching temperature of 90 °C. Planar or equibiaxial stretching of the set PET fiber web samples was performed to various stretch ratios at a stretching speed of 10 mm/min. The maximum stretch ratios for planar and equibiaxial stretching were MD (machine direction) × TD (transverse direction) = 4 × 1 (Figure 1c) and 4 × 4 (Figure 1d). The stretched samples were annealed under fixed-size condition by raising the temperature of the oven from 90 to 116 °C at a heating rate of approximately 3 °C/min and maintaining at 116 °C for 5 min.

### 2.4. Scanning Electron Microscopy (SEM)

The PET web samples were coated with Au via ion sputtering (E-1010, Hitachi Co. Ltd., Tokyo, Japan) and observed on a scanning electron microscope (SEM; VE-7800, KEYENCE Co. Ltd., Osaka, Japan). For each prepared PET web specimen, the diameters of 100 fibers were measured using an image analysis software (ImageJ version 1.52v), and the average diameter and coefficient of variation (CV) were obtained.

The distribution of the fiber orientation angle was analyzed from SEM images using a fiber orientation analysis software (Eizokun ver. 2.50, Asahi Kasei Engineering Co., Kawasaki, Japan).

Assuming that all fibers are aligned parallel to the MD–TD plane, a planar orientation factor, $f_{MD-TD}$, is defined by Equation (1):

(1) $$f_{MD-TD}=2\langle \cos^{2}\phi \rangle -1,$$

where ϕ is the fiber orientation angle with respect to MD, and $\langle \cos^{2}\phi \rangle$ is the mean value of $\cos^{2}\phi$ [29,30]. The $f_{MD-TD}$ values of 1 and −1 correspond to perfect orientation along the MD and TD, and the $f_{MD-TD}$ values of zero correspond to random orientation of fibers in the MD–TD plane.

### 2.5. Polarizing Microscope Analysis

The diameter and optical retardation of the fibers in the PET web samples were measured using a polarizing microscope (BX53-P, Olympus Co. Ltd., Tokyo, Japan) equipped with a polarizing filter and a Berek or Senarmont compensator [18]. The birefringence values, Δ*n*, were calculated using Equation (2):

(2) $$\Delta n=\frac{R}{d},$$

where *d* is the fiber diameter, and *R* is the optical retardation.

### 2.6. Differential Scanning Calorimetry (DSC)

Differential scanning calorimetry (DSC) was performed on the PET web and the film samples using a Q100 MI analyzer (TA Instruments Co, Inc., New Castle, DE, USA). The samples were measured in a temperature range between 25 and 300 °C at a heating rate of 10 K/min. Approximately 1 and 3 mg of the web and film samples were weighed for DSC measurements. The crystallinity of the samples was determined using the crystal melting endotherm, which was obtained by subtracting the exothermic heat of cold crystallization from the endothermic heat of melting. A heat of fusion of 140.1 J/g for 100% crystallinity was employed in the calculation of crystallinity [31].

### 2.7. Wide-Angle X-ray Diffraction (WAXD)

The WAXD measurements were performed using an X-ray generator (Rigaku Co., Akishima, Japan) and a CCD detector by generating an X-ray beam at a voltage of 45 kV and a current of 60 mA [18]. A Ni-filtered CuKα beam was used to obtain WAXD 2D intensity distributions for the PET web and film samples.

## 3. Results and Discussion

### 3.1. Fiber Diameters

**Figure 2:**
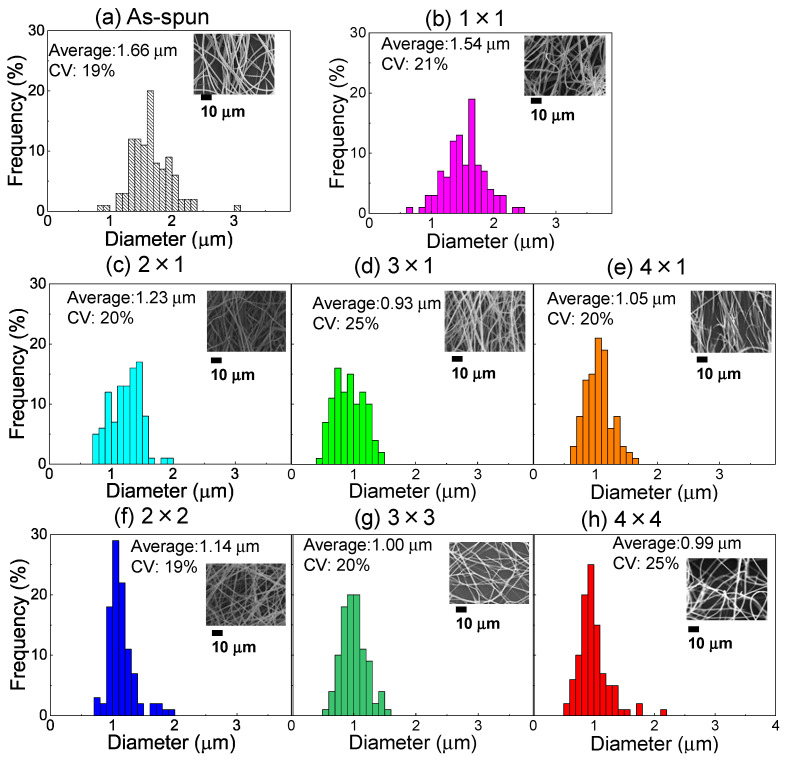
Diameter distribution of fibers in (**a**) the as-spun PET web and (**b**) the web after annealing (MD × TD = 1 × 1). The annealed webs after planar stretching at the stretch ratio of MD × TD = (**c**) 2 × 1, (**d**) 3 × 1, and (**e**) 4 × 1. The annealed webs after simultaneous equibiaxial stretching at the stretch ratio of MD × TD = (**f**) 2 × 2, (**g**) 3 × 3, and (**h**) 4 × 4. Average diameter and its coefficient of variation (CV) as well as the SEM image are shown for each web.

The fiber diameter distribution for the as-spun web sample and the web samples annealed at 116 °C for 5 min after planar stretching and simultaneous equibiaxial stretching at 90 °C are shown in Figure 2. The corresponding SEM images and the average and CV values of the diameter distribution are also shown in the figure. The relationship between the average fiber diameter of the unstretched web samples and annealed web samples after planar stretching and simultaneous equibiaxial stretching for various stretch ratios is summarized in Table 1. For both planar and equibiaxial stretching web samples, we found that the average fiber diameter decreased with an increase in the stretch ratio. The stretch ratio of fibers in the webs, estimated from the diameter changes, was lower than the applied stretch ratios, as shown in Table 1. When the stretch ratio exceeded MD × TD = 3 × 1 for planar stretching and MD × TD = 3 × 3 for equibiaxial stretching, the average fiber diameter hardly changed. Nevertheless, thinning of the fiber was achieved by applying the stretching process to the LES webs, and ultrafine fiber webs with an average fiber diameter of approximately 1 μm and a CV of 20–25% were obtained.

### 3.2. Fiber Orientation

**Figure 3:**
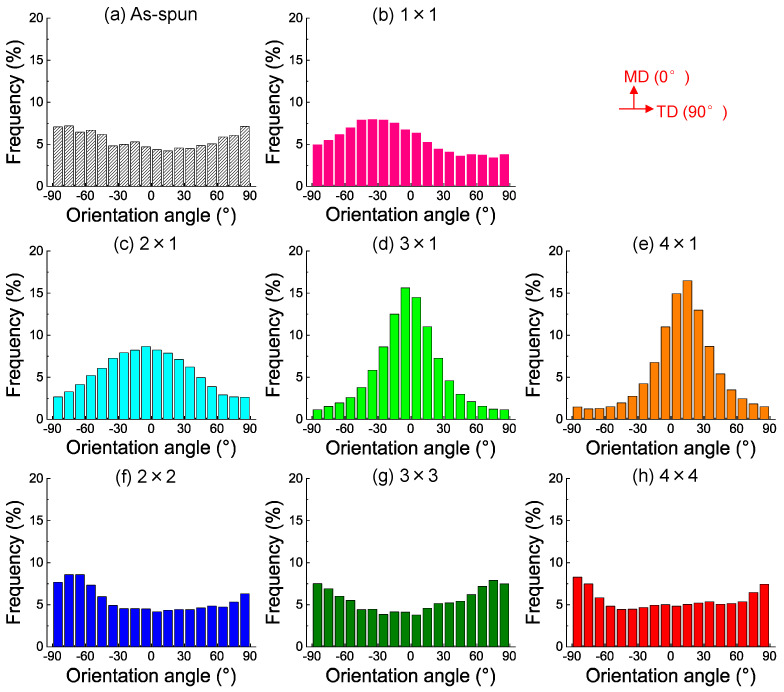
Orientation distribution of fibers in (**a**) the as-spun PET web and (**b**) the web after annealing (MD × TD = 1 × 1). The annealed webs after planar stretching at the stretch ratio of MD × TD = (**c**) 2 × 1, (**d**) 3 × 1, and (**e**) 4 × 1. The annealed webs after simultaneous equibiaxial stretching at the stretch ratio of MD × TD = (**f**) 2 × 2, (**g**) 3 × 3, and (**h**) 4 × 4.

The orientation distribution and planar orientation factors of the fibers in the webs analyzed from SEM images are shown in Figure 3 and Table 2. The planar orientation factors were calculated from the fiber orientation angles using Equation (1). In the case of planar stretching and subsequent annealing, the fibers were oriented towards the MD (orientation angle of 0°), which corresponds to the narrowing of the orientation distribution and increase in the planar orientation factor with an increase in the stretching ratio. In contrast, in the case of simultaneous equibiaxial stretching and subsequent annealing, the planar orientation factor was close to zero for all samples. This means that there was no preferential orientation of the fibers, and the web was isotropic in the web plane.

### 3.3. Observation under Polarizing Microscope

**Figure 4:**
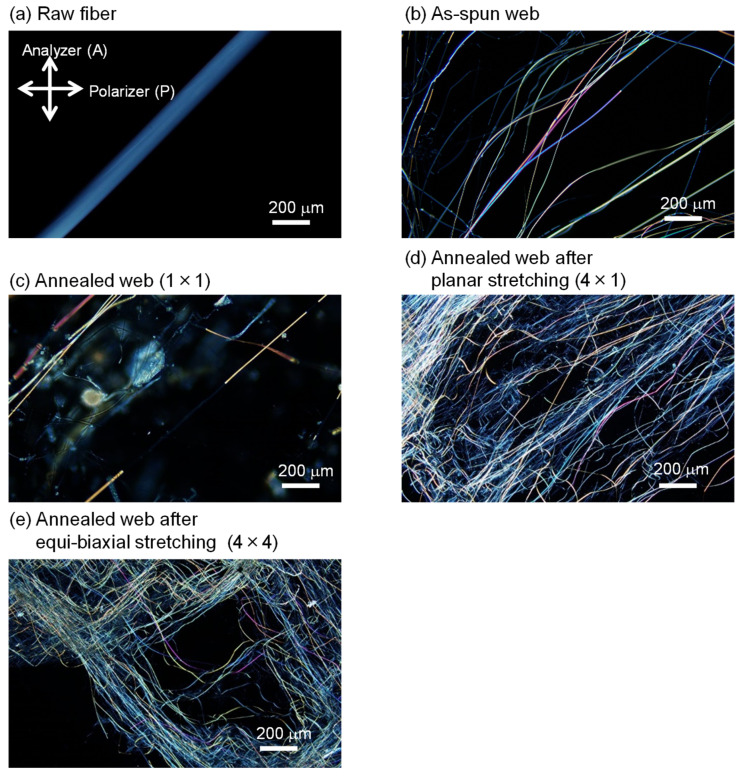
Micrographs of the fibers observed under a polarizing microscope with cross-polarization: (**a**) raw fiber for LES, (**b**) as-spun web, (**c**) annealed web (MD × TD = 1 × 1), (**d**) annealed web after planar stretching (MD × TD = 4 × 1), and (**e**) annealed web after simultaneous equibiaxial stretching (MD × TD = 4 × 4). Directions of polarization for a polarizer and an analyzer are indicated in (**a**).

**Figure 5:**
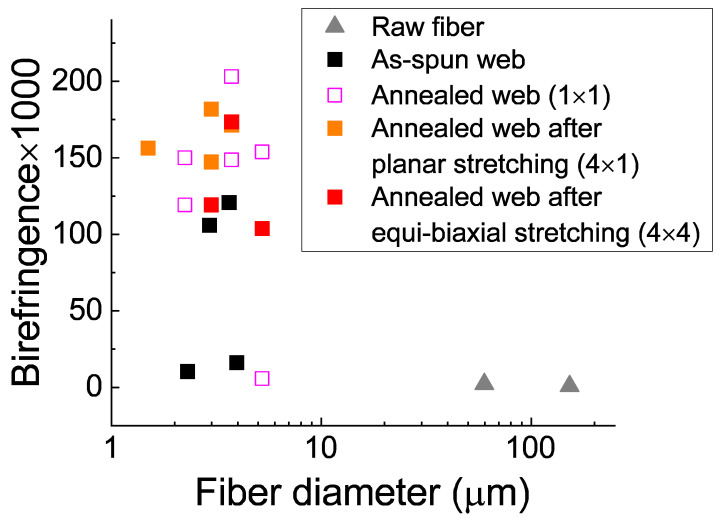
Correlation between fiber diameter and birefringence of a raw fiber and fibers in the web samples of various processing conditions.

Micrographs of the PET fibers observed under a polarizing microscope with cross-polarization are shown in Figure 4. The relationship between birefringence and diameter for various fibers is shown in Figure 5. Micrographs of raw material fibers and as-spun and annealed webs reported in a previous paper [18] are included in the figure for comparison purpose. Similar to the as-spun and annealed web samples [18], various interference colors are evident in the stretched and annealed webs under cross-polarization. The birefringence values of the fibers in the annealed webs after planar and equibiaxial stretching were approximately 147–172 × 10^−3^ and 104–174 × 10^−3^, respectively. These birefringence values are equivalent to the values reported for highly oriented yarns (HOY) [32]. In the as-spun web and annealed webs, a bimodal distribution of birefringence was observed [18]. Although the number of measurements was limited, it appears that the fibers with low birefringence were selectively stretched during planar and equibiaxial stretching because the birefringence of all the fibers in the stretched and annealed web were almost comparable with the higher birefringence values of the annealed web sample.

### 3.4. DSC Analysis

**Figure 6:**
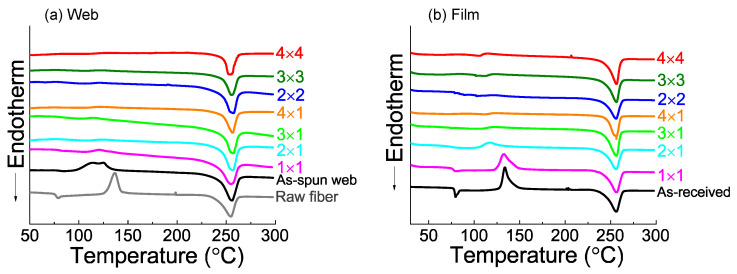
DSC thermograms of (**a**) web and (**b**) film samples of various processing conditions.

**Figure 7:**
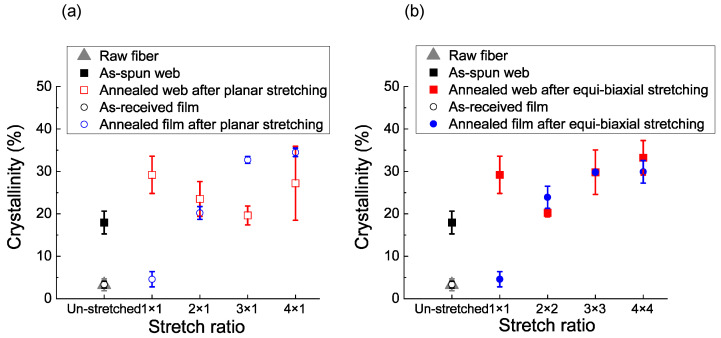
Crystallinity analyzed from DSC thermograms for the web and film samples prepared by annealing after (**a**) planar stretching and (**b**) simultaneous equibiaxial stretching. Data for the raw fiber for LES, as-spun web, and as-received film are also included.

The DSC thermograms of the various web and film samples are shown in Figure 6. The crystallinity values are shown in Figure 7. After applying planar or biaxial stretching and subsequent annealing to the web samples, the cold crystallization peak disappeared. In contrast, after applying planar or biaxial stretching and subsequent annealing to the film samples, the planarly stretched 2 × 1 film sample still exhibited a small cold crystallization peak at the lower temperature of around 117 °C as in the case of the annealed 1 × 1 film sample, in which cold crystallization peak was observed at around 132 °C. The difference between the web and film samples after stretching and annealing is attributable to the fact that the fiber in the as-spun web had a certain degree of molecular orientation compared to practically no orientation in the as-received film, which was confirmed from the lower cold crystallization temperature of the as-spun web compared to the as-received film.

Variation of crystallinity with stretching was analyzed from the results of DSC, as shown in Figure 7. For the web samples, the original 1 × 1 sample already exhibited a crystallinity of about 30%, and there was not much variation in crystallinity after applying planar or biaxial stretching. On the other hand, for the film samples, crystallinity suddenly increased by applying stretching to the original 1 × 1 sample, and there was an additional increase in crystallinity with the increase in stretch ratio. This behavior originated from the difference in the degree of orientation between the as-received film and as-spun fiber web samples, as discussed in the previous section.

### 3.5. WAXD Analysis

**Figure 8:**
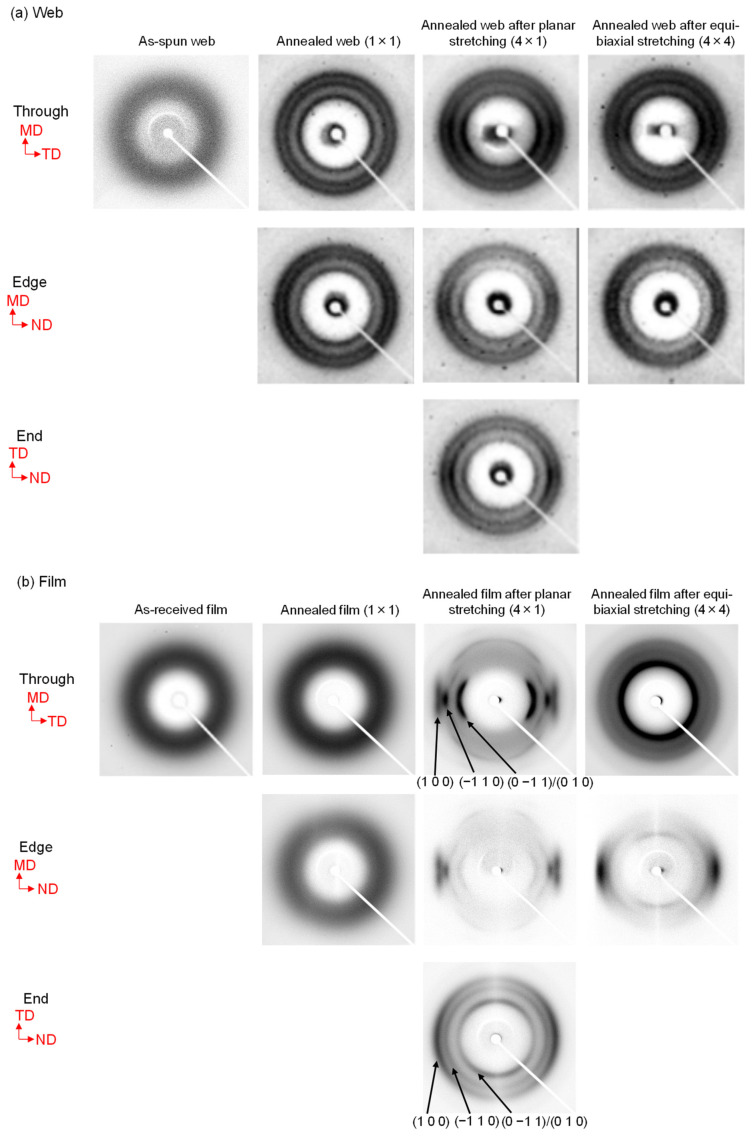
WAXD patterns of the (**a**) web and (**b**) film samples from various processing conditions obtained from the through, edge, and end directions. Machine direction (MD), transverse direction (TD), and normal direction (ND) are indicated in the figure.

**Figure 9:**
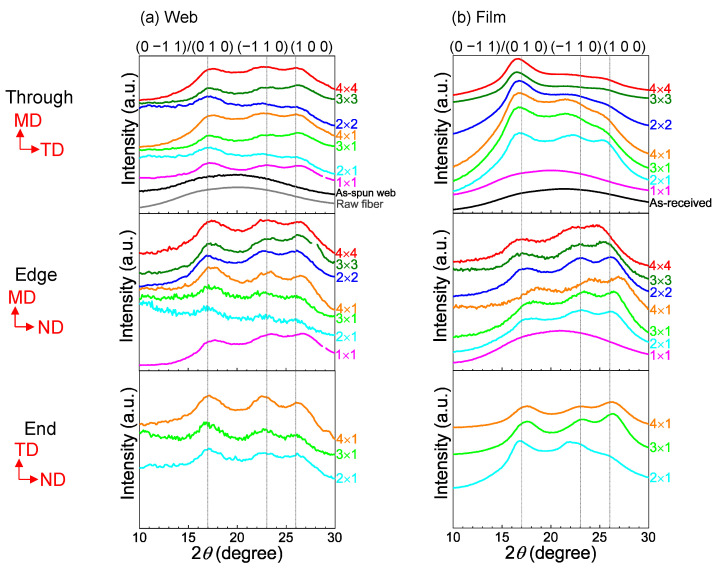
WAXD intensity profiles against diffraction angle for the (**a**) web and (**b**) film samples annealed after stretching to various stretch ratios. The intensity profiles were obtained by averaging the intensity along the azimuthal angle of 0 to 180°. Data for the as-received film, raw fiber for LES, and as-spun web are also included.

**Figure 10:**
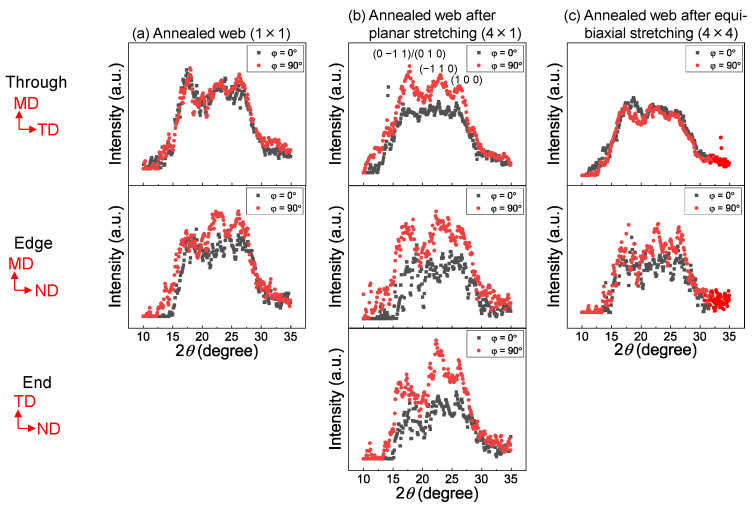
WAXD intensity profiles against diffraction angle for the azimuthal angles (φ) of 0 and 90° for the (**a**) annealed web (stretch ratio of 1 × 1), (**b**) annealed web after planar stretching (stretch ratio of 4 × 1), (**c**) annealed web after simultaneous equibiaxial stretching (stretch ratio of 4 × 4).

**Figure 11:**
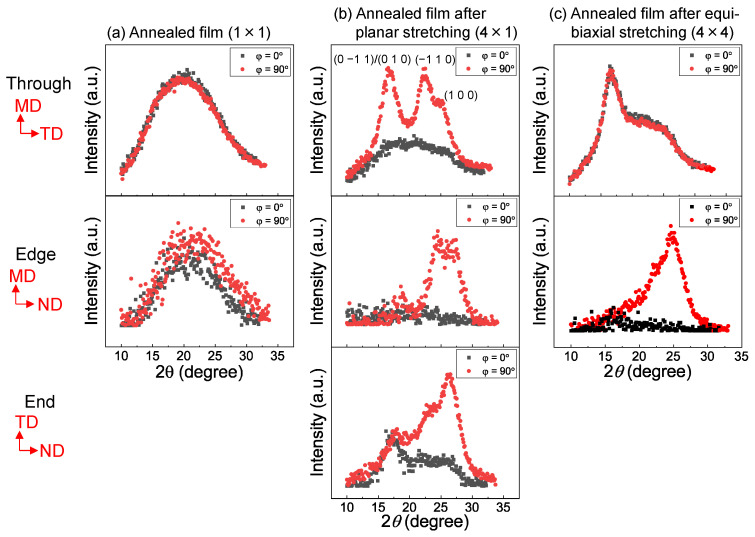
WAXD intensity profiles against diffraction angle for the azimuthal angles (φ) of 0 and 90° for the (**a**) annealed film (stretch ratio of 1 × 1), (**b**) annealed film after planar stretching (stretch ratio of 4 × 1), and (**c**) annealed film after simultaneous equibiaxial stretching (stretch ratio of 4 × 4).

The 2D WAXD patterns obtained from the through and edge directions of the web and film samples after stretching and annealing are shown in Figure 8. In this figure, the MD, TD, and ND correspond to the winding/machine direction, transverse direction, and normal direction, respectively. The WAXD intensity profiles of the annealed web and film samples of various stretch ratios obtained by averaging the intensity along the azimuthal direction from 0 to 180° are shown in Figure 9. Data for the web and film samples without stretching and the raw fiber for LES, which were reported in a previous paper [18], are also included for comparison purpose in Figure 8 and Figure 9. The intensity profiles at azimuthal angles (φ) of 0 and 90° for the stretched web and film samples, including the annealed 1 × 1 samples for comparison [18], are presented in Figure 10 and Figure 11, respectively. The intensity peaks at approximately 2θ = 17, 23, and 26° correspond to the (0–11)/(010), (–110), and (100) reflections, respectively, of the triclinic crystalline unit cell for PET [33].

As reported in a previous paper [18], for all the unstretched samples, that is, as-spun (unstretched) web prepared by LES, melt-spun fiber for LES, and as-received film, only an amorphous halo was evident in both the 2D WAXD patterns and WAXD intensity profiles. For samples with a stretch ratio of 1 × 1 (no stretch) and annealing temperature and period of 116 °C and 5 min, respectively, only an amorphous halo was apparent in the through and edge views of the film samples, while the crystalline reflections of isotropic rings appeared in the through and edge views of the web samples.

The characteristics of the WAXD patterns of the stretched and annealed web and film samples shown in Figure 8 were as follows. It is known that stretched PET film exhibits unique preferential orientation of benzene ring along the film plane [19,20,21,22,23,24,25,26,27,28,29,30,31]. For PET film samples annealed at 116 °C for 5 min after planar stretching at 90 °C, (100) reflections were weaker compared to (0–11) and (010) reflections in the through view, (100) reflections were stronger compared to (0–11) and (010) reflections in the edge view, and (−110) and (100) were evident in the ND whereas (0–11)/(010) were evident in the TD in the end view. For the PET film samples annealed at 116 °C for 5 min after equibiaxial stretching at 90 °C, (0–11)/(010) selectively appeared in the through view, while (−110) and (100) appeared in the ND in the edge (and end) view(s). These were typical characteristics of WAXD patterns for stretched PET films in that (100), which contains a benzene ring, selectively aligned along the film plane. On the other hand, for the PET web samples, despite applying planar or equibiaxial stretching at 90 °C and subsequent annealing at 116 °C for 5 min, selective intensity variation of each crystalline reflection, as observed in the through, edge, and end views for film samples, was not detected. This implies that axial symmetry around the c-axis oriented along the web plane was maintained, and there was no tendency of uniplanar–axial orientation.

A similar tendency can be confirmed in Figure 9, where the degree of uniplanar–axial orientation appears to increase with an increase in the stretch ratio for both planar and equibiaxial stretching of films. No change in the ratio of intensity of crystalline planes was evident in the stretched and annealed web samples. As reported in a previous paper [18], after annealing at 116 °C, the web sample crystallized while the film sample remained in an amorphous state.

To clarify the effect of orientation on the WAXD patterns, diffraction intensities at azimuthal angles of 0 and 90° in the through, edge, and end views are compared in Figure 10 and Figure 11 for the web and film samples, respectively. For the annealed film samples, the unstretched film was still in an amorphous state without any orientation, whereas the film equibiaxially stretched at 90 °C and subsequently annealed at 116 °C for 5 min showed a crystalline structure with a uniplanar orientation of (100) in the film plane. This was confirmed by the disappearance of the (0–11)/(010) reflections in the edge view and the (100) reflection in the through view. The film planarly stretched at 90 °C and subsequently annealed at 116 °C for 5 min was expected to exhibit a uniplanar–axial orientation where the c-axis was oriented to MD. The stronger intensity of the (−110) reflection in the through view and the appearance of the (0–11)/(010) reflection in the end view suggested that the degree of uniplanar orientation along (100) was weaker compared to the equibiaxially stretched and subsequently annealed film, especially for the molecules oriented perpendicular to the MD.

Similar to the annealed web (unstretched: 1 × 1) samples, the webs annealed after planar and equibiaxial stretching showed a crystalline structure with a mild c-axis orientation along the web plane. This was confirmed by the slightly higher equatorial intensity than the meridional intensity in the edge view. From the through views, it could be observed that the crystalline c-axes were randomly oriented in the web plane of the web equibiaxially stretched at 90 °C and subsequently annealed at 116 °C for 5 min, whereas the c-axes were uniaxially oriented along the MD in the web planarly stretched at 90 °C and subsequently annealed at 116 °C for 5 min. The orientation distribution of the fiber axis was evidently narrower in the MD–ND plane than in the MD–TD plane. There was no indication of the uniplanar orientation along (100) in the web samples.

In the case of applying stretching and subsequent annealing to the webs prepared by LES, it should be noted that the crystalline orientation in the obtained webs can be affected by two major factors, i.e., the degree of orientation of individual fibers in the web and the degree of orientation of crystallites in individual fibers.

In the webs after planar stretching, the fibers in the web were oriented towards the MD, and the molecules and crystallites in the fiber were uniaxially oriented along the fiber axis. The orientation distribution of the fiber axis was evidently narrower in the MD–ND plane than in the MD–TD plane, whereas crystallites in the individual fibers were expected to exhibit uniaxial orientation. On the other hand, in the webs after simultaneous equibiaxial stretching, there was no preferential orientation of the fibers in the web plane, but the orientation of fibers along the web plane as well as the orientation of molecules and crystallites in the individual fibers along the fiber axis were promoted as in the planarly stretched web samples. Independent evaluation of the degrees of orientation of the fiber axis in the web and the molecular chain axis in the individual fiber is an important subject that needs to be studied in future work.

## 4. Conclusions

PET fiber webs were prepared through laser-heated electrospinning (LES) and subsequent planar or simultaneous equibiaxial stretching processes. We found that for both planarly and equibiaxially stretched web samples, the average fiber diameter decreased with an increase in the stretch ratio until MD × TD was 3 × 1 and 3 × 3 for planar and equibiaxial stretching, respectively. Ultrafine fiber webs with an average fiber diameter of approximately 1 μm and a CV of 20–25% were obtained.

In terms of fiber orientation, in planar stretching, the fibers were oriented towards the stretching direction and narrowing of the orientation distribution and increase in the planar orientation factor were confirmed. On the other hand, in simultaneous equibiaxial stretching, there was no preferential orientation of the fibers and the web was isotropic in the web plane.

Investigation on the structure of fibers in the stretched and annealed webs revealed that the fibers had high birefringence, equivalent to the values reported for highly oriented yarns (HOY). The DSC measurements showed that the crystallinity of the web sample hardly changed with the stretch ratio, while the crystallinity of the film sample increased with increasing stretch ratio. The WAXD results indicated that the films after planar or simultaneous equibiaxial stretching exhibited preferential orientation of the (100), which includes a benzene ring in the main chain of PET, along the film plane. However, for the annealed webs after planar or simultaneous equibiaxial stretching, although preferential orientation of the molecular chain along the web plane was confirmed, no preferential orientation of the crystallographic plane was observed. It was also noted that the orientation distribution of the fiber axis for planar stretching was narrower in the MD–ND plane than in the MD–TD plane.

The results obtained reveal that further thinning and structural development of PET fibers in webs prepared through LES can be accomplished through planar or biaxial stretching and annealing processes. Because PET fibers in the web are expected to have high mechanical properties and high thermal stability, fabricated webs have potential for a wide variety of applications, such as packaging and battery separator materials.

## Figures and Tables

**Table 1:** Diameter and stretch ratio of the fibers in the stretched and annealed webs of various stretch ratios.

No.	Unstretched (As-Spun) Web		Stretched and Annealed Web			
	Fiber Diameter		Stretch Ratio: MD × TD	Fiber Diameter		Stretch Ratio of Fiber in Web
	Average (μm)	CV (%)		Average (μm)	CV (%)	
1	1.66	19	1 × 1	1.54	21	1.16
2	1.38	17	2 × 1	1.23	20	1.26
3	1.43	12	3 × 1	0.93	25	2.36
4	1.65	22	4 × 1	1.05	20	2.47
5	1.56	11	2 × 2	1.14	19	1.87
6	1.46	13	3 × 3	1.00	20	2.13
7	1.61	24	4 × 4	0.99	25	2.64

**Table 2:** Fiber orientation factor in the webs.

No.	Stretch Ratio: MD × TD	Planar Orientation Factor: $f_{MD-TD}=2\langle \cos^{2}\phi \rangle -1$
0	Unstretched (as-spun)	−0.0669
1	1 × 1	0.0117
2	2 × 1	0.2532
3	3 × 1	0.5515
4	4 × 1	0.4905
5	2 × 2	−0.0855
6	3 × 3	−0.0968
7	4 × 4	−0.1452

## Data Availability

Not applicable.
